# A Rare Location of Metastasis from Prostate Cancer: Hydronephrosis Associated with Ureteral Metastasis

**DOI:** 10.1155/2012/656023

**Published:** 2011-09-07

**Authors:** Sebastian Schneider, Dieter Popp, Stefan Denzinger, Wolfgang Otto

**Affiliations:** Department of Urology, St. Joseph's Mecial Center, University of Regensburg, Landshuter Straße 65, 93053 Regensburg, Germany

## Abstract

Prostate carcinoma is a very rare origin of metastatic disease in the ureter. We report a case of a 74-year-old man who presented in November 2008 initially with flank pain and lower urinary tract symptoms. Diagnostic investigation revealed a skeletal metastasizing prostate carcinoma, and the cause for the flank pain was a hydronephrosis due to ureteral metastasis diagnosed by biopsy. Antihormonal treatment led to disappearance of the hydronephrosis; however, further progress finally ended in acute liver failure with patient's death in July 2010.

## 1. Introduction

The ureter is a rare location of metastasis for primary tumors of any kind. In 1909, Stow described the first case of a truly metastatic ureteral lesion from a lymphosarcoma [1]. The most common malignant tumors metastasizing to the ureter are breast cancer followed by stomach cancer and colorectal cancer. Only 43 cases of metastasis in the ureter by primary adenocarcinoma of the prostate have been reported during the last century [2–7]. We present the first case of such a patient since the last 2 years.

## 2. Case Report

A 74-year-old man in November 2008 first presented at an outpatient urologist, due to lower urinary tract symptoms and intermittent pain of the right flank. The digital rectal examination revealed an enlarged, palpatory suspicious prostate gland with an endured left lobe. The PSA level was initially at 52 ng/mL. The subsequent prostate biopsy showed a low differentiated adenocarcinoma of the prostate gland (Grade 2, Gleason score 7 = 3 + 4). Further diagnostics with bone scan and ultrasound provided the evidence of diffuse skeletal metastasis as well as an hydronephrosis of the right kidney. Because of the findings, an antihormonal therapy with LHRH-analogues was initiated.

**Figure 1:**
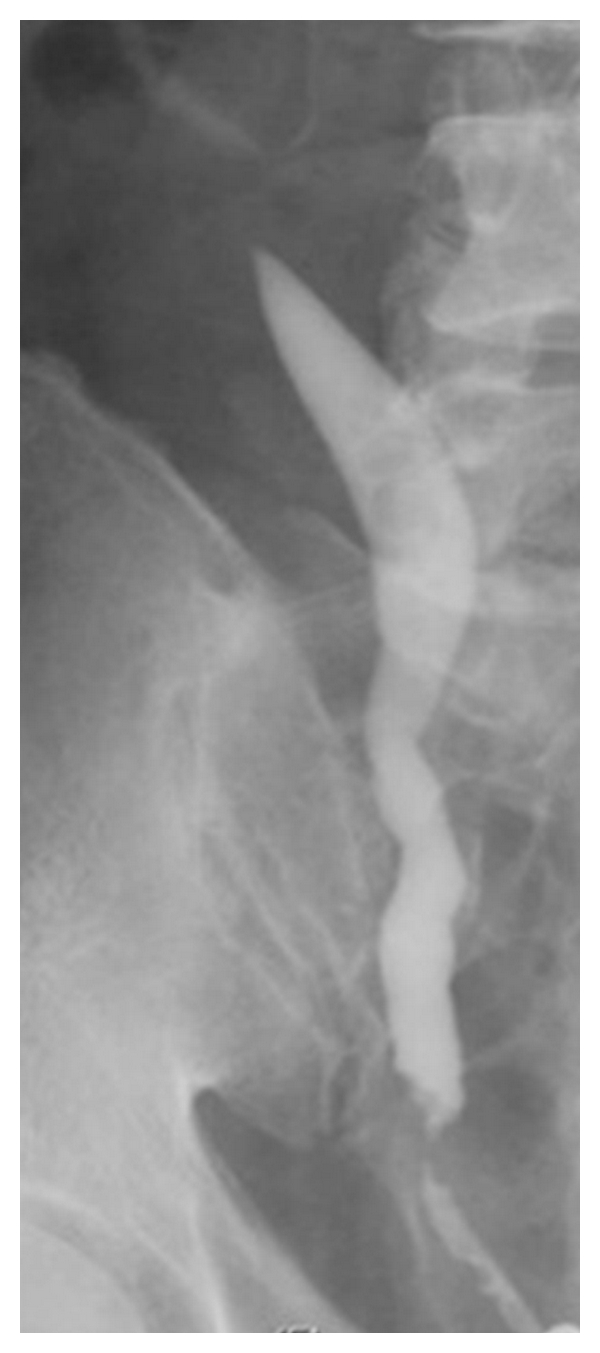
Retrograde pyelography showing an obstruction of the lower part of the right ureter.

**Figure 2:**
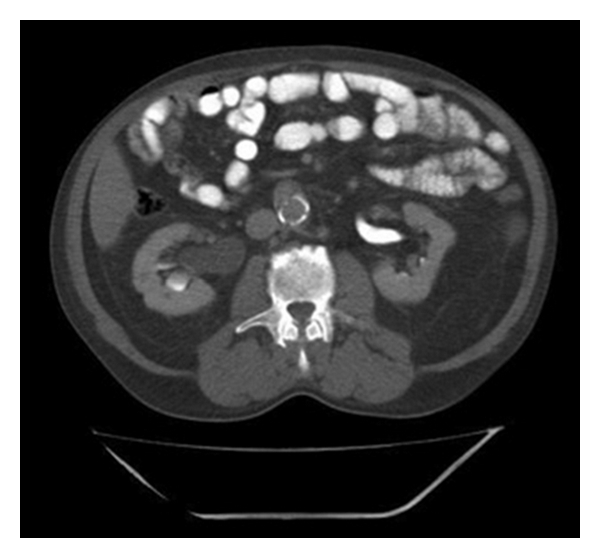
Computer tomography with hydronephrosis of the right kidney.

**Figure 3:**
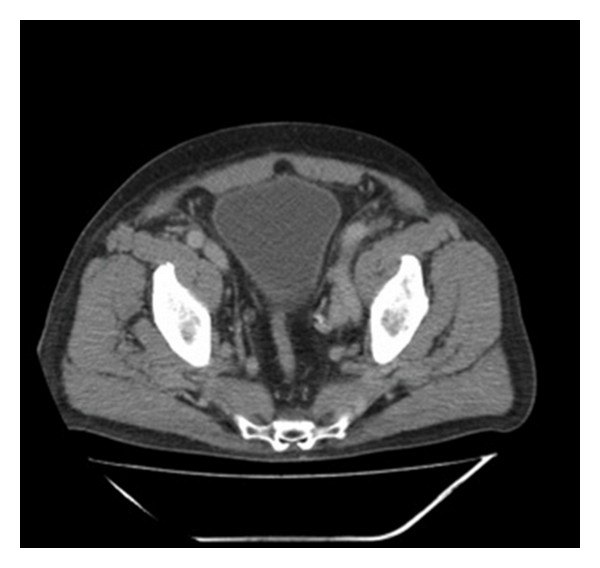
Presentation of an intraluminal ureteral mass of the right upper urinary tract in computer tomography of the pelvis.

As further diagnostics, an intravenous pyelography showed a delayed excretion of the right kidney and a significant hydronephrosis with a dilated ureter. Cause of the prestenotic dilatation was a stricture of the lower part of the ureter (Figure 1). Due to the unclear nature of the stricture, a computer tomography was performed, detecting a suspicious intraluminal ureteral mass at the height of the bifurcation of the A. iliaca (Figures 2 and 3). An extraluminal compression of the ureter, as shown with lymph nodes or other solid masses, could not be demonstrated. In addition, there was no evidence of suspicious enlarged subdiaphragmal or pelvic lymph nodes. The blood and urine counts were except for a microscopic hematuria inconspicuous. Ureterorenoscopy with biopsy of the ureteral mass was the next step in the diagnosis of the patient. In ureterorenoscopy, the reported intraluminal mass was found in the distal ureter, as well as in the middle ureter. After collecting urine for cytology, each of the intraluminal lesions underwent biopsy. Histology by immunohistochemical analysis revealed a metastasis of the ureter by a prostate adenocarcinoma. Cytology did not reveal pathological result.

Because of the symptomatic hydronephrosis and suspected ureteral tumor, we first placed a nephrostomy after ureterorenoscopy. Postoperatively, there were no complications, but at the fourth postoperative day, the nephrostomy dislocated. Since then, however, no relevant hydronephrosis existed and the patient remained pain-free, so that no further insertion of a nephrostomy was indicated.

The following procedure in case of multiple skeletal metastasis by prostate cancer was a continuation of the therapy with LHRH analogues in combination with zoledronic acid infusions once a month. In June 2009, PSA progress led to maximum antihormonal therapy with LHRH analogues and antiandrogene. Further progress in January 2010 indicated docetaxel chemotherapy that was prematurely stopped by appearance of liver metastases. In July 2010, the patient died of acute liver failure without having experienced further hydronephrosis.

## 3. Discussion

In 1999, Haddad described over all 38 cases of prostate carcinoma with at least one ureteral metastasis. For this paper, the authors considered data from the last century until 1987 [2]. For example, McLean showed 1956 of 10,223 cancer patients with only 18 cases of ureteral metastasis, and even only one of them related to a prostate carcinoma [8]. Kirshbaum et al., 1933, also demonstrated the rarity of ureteral metastasis by a series of 4,860 autopsies. They found in these patients only 5 ureteral metastasis, and only 2 of them were metastatic from adenocarcinoma of the prostate gland [9]. In a case report, Hulse and O'Neill described a true ureteral metastasis of a prostate carcinoma associated with a ureteral stone [3]. Cohen et al. underlined in 1974 the uncommon way of metastasis to the ureter from a prostate carcinoma. They showed 3,200 autopsies with 31 cases of ureteral metastasis but none of them with a prostate cancer as origin [10]. Singh et al. presented in 2009 a case report of a man with a locally advanced prostate cancer and bone metastasis with ureteral metastasis in both ureters [4]. In 2007, Marzi described another case with undifferentiated prostate cancer and neoplastic metastasis of prostatic origin in both ureters too [5]. Jung et al. showed a ureteral metastasis from prostate adenocarcinoma after bilateral orchiectomy in 2000, and Yonneau et al. presented in 1999 a patient with an episode of renal colic and a ureteric tumor with a history of prostatectomy for prostate cancer. They performed ureterectomy, and histology examination revealed a metastasis from prostatic adenocarcinoma to the ureter [6, 7]. Altogether, only 43 cases of an adenocarcinoma of the prostate metastatic to the ureter have been reported in the last century [2–7]. In the first half of the last century, these ureteral metastases have been only developed by incidental finding during autopsy. Later ultrasonography and ureteroscopy have been established, so that also clinical cases were reported. However, only a few patients with ureteral metastasis are identified, because up to 85% patients are staying asymptomatic [3]. In case of disorders, the most frequently is flank pain (15–50%), hematuria is rather rare (16%) [3, 11].

In our case, the patient complained intermittent right flank pain together with a hydronephrosis of the right upper urinary tract. During medication with LHRH analogue, hydronephrosis disappeared completely.
